# Does an in-Season 6-Week Combined Sprint and Jump Training Program Improve Strength-Speed Abilities and Kicking Performance in Young Soccer Players?

**DOI:** 10.2478/hukin-2013-0078

**Published:** 2013-12-31

**Authors:** Mário C. Marques, Ana Pereira, Ivan G. Reis, Roland van den Tillaar

**Affiliations:** 1 Department of Sport Sciences, University of Beira Interior, Covilhã, Portugal.; 2 Research Centre for Sport, Health and Human Development, Vila Real, Portugal.; 3 Department of Teacher Education and Sports of Sogn and Fjordane University College, Norway.; 4 Department of Sport Sciences, University of Trás-os-Montes and Alto Douro, Vila Real, Portugal.; 5 Department of Sport Sciences, University of Pará, Belém, Brasil.

**Keywords:** speed training, lower extremity, kicking, soccer

## Abstract

The aim of this study was to examine the effect of a six-week combined jump and sprint training program on strength-speed abilities in a large sample of youth competitive soccer players. It was hypothesized that the experimental training group would enhance their jumping and sprinting abilities. Enhancement of kicking performance was also hypothesized due to an expected increase in explosive strength established by a plyometric and sprinting regimen. Fifty-two young male soccer players playing at the national level (aged 13.4 ± 1.4 years, body mass 53.4 ± 11.7 kg, body height 1.66 ± 0.11 m) took part in the study. Half of the group underwent the plyometric and sprint training program in addition to their normal soccer training, while the other half was involved in soccer training only. The plyometric training group enhanced their running (+1.7 and +3.2%) and jumping performance (+7.7%) significantly over the short period of time, while the control group did not. Furthermore, both groups increased their kicking velocity after just six weeks of training (+3.3 vs. 6.6%). The findings suggest that a short in-season 6-week sprint and jump training regimen can significantly improve explosive strength in soccer-specific skills and that these improvements can be transferred to soccer kicking performance in terms of ball speed.

## Introduction

Soccer is the world’s most popular sport. Due to this fact, many studies have been conducted in an attempt to provide understanding of the essential skills required by players (Cometi et al., 2001; Wisloff et al., 2004; Chelly et al., 2009; Mújica et al., 2009; Requena et al., 2009). Unfortunately, there is a relatively small body of scientific knowledge in this field, and consequently most players acquire soccer skills as a result of individual training experiences rather than academic research-based instruction (López-Segovia et al., 2011).

Running is a predominant activity involved in playing soccer, while explosive-type activities such as sprinting, jumping, tackling and kicking are important factors for successful performance (Wisloff et al., 1998; Hoff and Helgerud, 2004; Thorlund et al., 2009). Although explosive performance has been studied in soccer (Wisloff et al., 1998; Haff et al., 2001; Wisloff et al., 2004), only few studies have investigated the effects of plyometric training programs on youth competitive soccer players. The reason why activities such as plyometrics (for example, jumping exercises) are so effective in improving speed is that they cannot be performed without a high rate of power production, the rapid application of force, and acceleration. This is precisely why plyometrics correspond dynamically with many athletic activities involved in soccer, such as jumping, sprinting and kicking, and thus, should be assigned a high level of priority in training (Chamari et al., 2008).

As far as the effect of plyometric training on motor performance is concerned, research has mainly focused on determining the influence of different plyometric programs on jumping (Diallo et al., 2001; Christou et al., 2006; Michailidis et al., 2012) and sprinting performance rather than kicking. Although some authors have identified significant relationships between the strength of the lower limbs and ball-kicking velocity in soccer players (Vera et al., 2008), there is still a lack of data regarding the effects of a plyometric training program on the characteristics of an activity such as kicking, especially in youth competitive male soccer players. Indeed, with regard to male players the effects of strength training on kicking performance are elusive. Manolopoulos et al. (2004), Perez-Gomez et al. (2008) and Sedano Campo et al. (2009) concluded that strength training focusing on the lower limbs can lead to improved kicking performance in soccer in terms of ball speed, but it takes time to transfer this to a specific activity. Several authors (Billot et al., 2010; Anderson and Dorge, 2011) have stated that the discrepancy between the findings of previous experiments may be explained by different research protocols such as differences in the length of training programs, the status of subjects, and training loads.

To the best of our knowledge, modifications in jumping, sprinting and kicking performance as a result of a plyometric training regimen have not been investigated simultaneously as part of a study involving a large number of young competitive soccer players. Therefore, the aim of this study was to examine the effect of a six-week combined plyometric and sprint program on different motor abilities and kicking speed in youth soccer players. It was hypothesized that the training group would enhance their jumping and sprinting performance. Enhancement of kicking performance was also hypothesized due to an expected increase in explosive strength established by the plyometric and sprinting program.

## Material and Methods

### Experimental Approach to the Problem

A repeated-measures design involving two groups (a training group and a control group) was used in order to determine the effectiveness of a plyometric and sprint training program on different motor abilities and kicking skills of youth soccer players as part of a 6-week training program. A randomized controlled study was conducted involving three teams of youth male soccer players. The three teams all had the same number of members at the pretest stage. Half of each team underwent the plyometric and sprint program in addition to normal soccer training and the other half was involved in soccer training only.

### Subjects

Fifty-two competitive youth male soccer players (aged 13.4 ± 1.4 years, body mass 53.4 ± 11.7 kg, body height 1.66 ± 0.11 m) took part in the study. Participants belonged to three different teams playing at the national level in their age category. Subjects were fully informed about the protocol before the start of the study. Informed consent was obtained prior to testing from all subjects and parents in accordance with the recommendations of the local ethical committee and current ethical standards in sports and exercise research.

### Procedures

Before the pretest stage the participants were familiarized with the different tests during a practice session in order to avoid the learning effect. Pre- and post-tests were performed with maximal intensity. All tests were conducted in an indoor facility in order to eliminate the effect of weather conditions on results.

After a general warm-up of 15 minutes, each participant was tested for explosive strength of the lower limbs by means of a counter movement jump (CMJ) (Wisloff et al., 2004). Participants started from a standing position with their hands on their waist, standing on a contact mat (Ergojump, 1000 Digitime, Digest, Finland). Next, they flexed their knees to 90°, and then jumped as high as possible while holding their hands on their waist. Flight time was measured and the jump height was calculated from flight time. Three attempts were made, with 2 minutes of rest between them.

Subjects were required to perform three 30 m sprints. Times at 0–15m (T_15_), 15–30m (T_15–30_) and 0–30m (T_30_), were recorded using photocells (Brower Wireless Sprint System, USA). Sprints were separated by 3 minutes of rest. The best attempt was considered for further analysis.

Finally, maximal kicking velocity was evaluated by players kicking a standard soccer ball (mass approximately 430g, circumference 70 cm) straight forward as hard as possible over a 25 m distance. The maximal kicking velocity of the ball was determined using a Doppler radar gun (Sports Radar 3300, Sports Electronics Inc.), with ± 0.028 m·s^−1^ accuracy within a field of 10 degrees from the gun. The radar gun was located 1 m behind the goal at ball height during the kick. Two minutes of rest was allowed between each attempt. Three attempts were made and the best one was recorded.

After the pretest, participants from each team were randomly divided into a training group (n=26) and a control group (n=26). The training group conducted an additional short plyometric and sprint training program consisting of four jumping exercises per session followed by sprint drills (Table 1).

**Figure 1:**
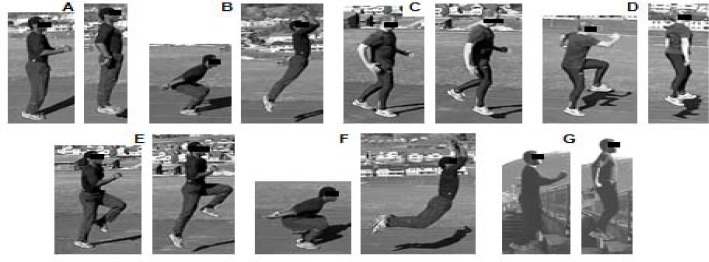
*Jumping exercises.*
2-legged jumps2-legged jumps as high as possible (knees bent)Short, quick hops on one legHeading without ball1-legged jumps as high as possible2-legged jumps as far as possible (knees bent)2-legged jumps up steps

The jumping exercises focused on limited ground contact, which is important for increasing explosive power of the lower limbs (Billot et al., 2010). A full description of the exercises is contained in Figure 1. The training load was increased in accordance with the principle of overload (Chelly et al., 2009). Each participant repeated the training program twice a week for 6 consecutive weeks. Participants were subject to this training besides their regular soccer training, which consisted of four sessions per week. The length of the strength-speed training program was 20 minutes. A coach with several years of experience in plyometric and sprint training supervised each training session in order to ensure that participants performed the exercises with maximal intensity and proper form. The control group was involved in a regular training regime only for the duration of the experiment.

### Statistical Analysis

A one-way ANOVA was performed on the anthropometric variables and considered motor abilities and skills (sprinting, jumping and kicking) of the two groups at the pre-test stage. In order to compare the effects of the training protocol, a mixed design 2 (test occasion: pre-post: repeated measures) × 2 (group: training vs. control group) analysis of variance (ANOVA) was carried out. Test-retest reliability (3 repeats per condition) as indicated by intra-class correlations (ICC) was 0.95, 0.87, 0.97, 0.95, and 0.90 respectively for peak-kicking ball velocity, running times (0–15m, 15–30m and 0–30m) and jump height. The level of significance was set at p ≤ 0.05.

## Results

**Figure 2:**
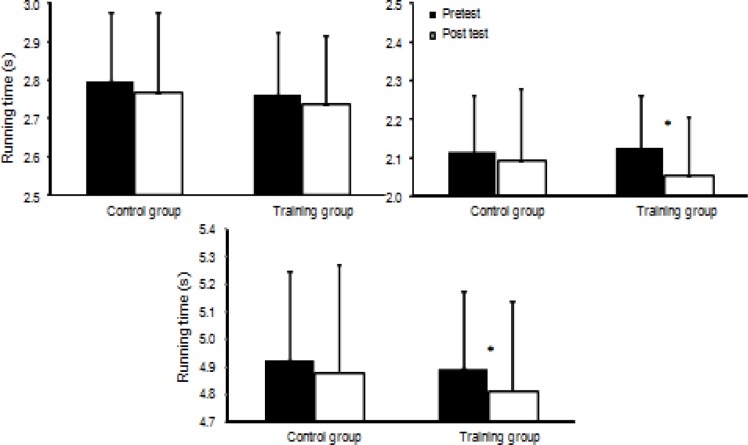
Running times (Mean ± SD) at 15m, 30 m and between 15 and 30 m, for the training and control groups. (*) indicates significant main effect from pre- to post test for this group (p<0.05).

**Figure 3:**
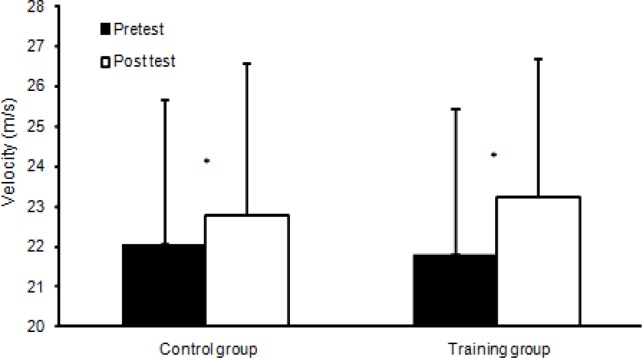
Peak ball velocity (Mean ± SD), for the training and control groups. (*) indicates significant main effect from pre- to post test for this group (p<0.05).

**Figure 4:**
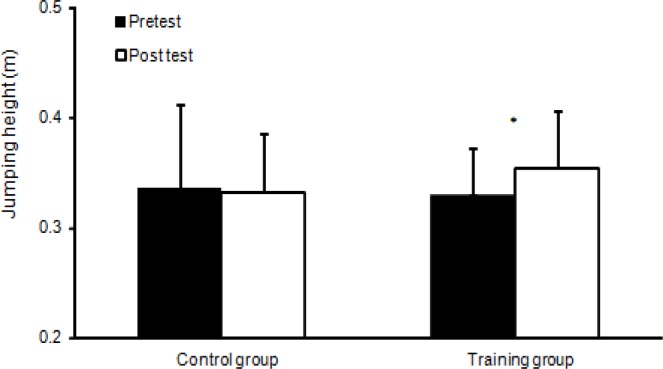
Jump height (Mean ± SD), for the training and control groups. (*) indicates significant main effect from pre- to post test for this group (p<0.05).

Pretest data indicated no statistical differences (p = 0.42) in term of anthropometrics or performance between the two groups. A significant increase was found following the training period in term of running times (p ≤ 0.026; Figure 2) and kicking velocity (p<0.001; Figure 3). No significant difference was found in terms of jump height after the training period (p=0.12). However, an interaction effect (p=0.036) between the groups in terms of jump height was found; the training group increased their jump height significantly (+7.7%), while the performance of the control group remained unchanged (−1.1%; Figure 4). No significant interaction effect (training group) was found for running times (p=0.992: 0–15m, p=0.058: 15–30m, p=0.297: 0–30m) or kicking velocity (+6.6: training group vs. +3.3%: control group; p=0.07). However, post hoc analysis showed that the running time of the training group decreased significantly between 15–30m (+3.2%) and 0–30m (+1.7%; p < 0.001; Figure 2) while the control group did not show any significant changes in running times (+0.9%; p ≥ 0.14; Figure 2).

## Discussion

The aim of this study was to examine the effect of a six-week combined plyometric and sprint program on strength-speed abilities and kicking velocity in youth soccer players. The main finding is that the training group enhanced their running and jumping performances significantly over a short period of time, while the control group did not. Furthermore, both groups increased their kicking velocity after just six weeks of training. However, the difference in kicking velocity between the two groups was not significant.

Vertical jump height increased for the experimental group only (+7.7%) while no significant changes were found in the control group (−1.1%), what is in accordance with the findings of Sedano et al. (2009), who also found an increase of 8.5% in jump height after 6 weeks of plyometric training in female soccer players. The improvement in jump height indicates that adaptations associated with increases in leg muscle power have occurred. Adaptations to training are likely to be neural because these predominate in the early stages of strength and power training (Billot et al., 2010) and have been shown to be the main adaptation to plyometric exercise (Diallo et al., 2001; Michailidis et al., 2012). Other factors may have contributed to the improvement in vertical jump performance in the experimental group, including improved synchronization of body segments, increased coordination levels, and greater muscular strength. These factors may be related to a more effective skill domain in vertical jumping, also contributing to explaining the lack of improvement in the control group. After 8 weeks of training of low intensity, Diallo et al. (2001) also found significant improvements in jumping (p<0.01) ability using a similar plyometric training regimen in youth male soccer players. During this period, no significant performance increase was recorded in the control group. These results demonstrate that short-term plyometric training programs increase athletic performance in prepubescent boys. In contrast, Chimera et al. (2004) did not find any significant increase in jumping ability after a 6-week plyometric training program. Similarly, Alves et al. (2010) failed to observe any significant change in CMJ performance in youth athletes after complex and contrast training. The difference in the frequency of training could be the reason for the discrepancy in the results. Alves et al. (2010) considered that these trends were related to the fact that the training program included only one training session per week. According to these authors, improving jump performance would require a minimum of two training sessions per week. However, in the present study, the use of two training sessions per week produced significant increases in CMJ height. Moreover, many authors have suggested that muscular performance gains after plyometric training are attributable to neural adaptations rather than morphologic changes (de Villarreal et al., 2008). According to these authors, neuromuscular factors such as increasing the degree of muscular coordination and maximizing the ability to use the muscles’ stretch-shortening cycle appear to be more important than changes in fiber size. The findings of the present study are in agreement with these authors because there were no interaction or time effects with regard to the muscle mass component. Nevertheless, neither muscle mass nor neuromuscular variables were assessed in the present study. Further studies focusing on neuromuscular factors are required in order to corroborate this for soccer players. The improvement of muscular coordination following the training period is probably partly related to the specificity of movements used during the training program (Davids et al., 2008).

Sprint times only decreased significantly in the training group between 15 and 30m, and over the total 30m (produced by a faster time between 15 and 30m), but not in the first 15m. An explanation for this could be that most jump exercises in the program in this study focused on vertical force and limited ground contact. This enhances vertical strength and power in participants. As shown by Mann (2011), in sprinting, vertical force is of key importance after the first 10m of a sprint start. In the first few meters after the start, horizontal force is more important (Zatsiosky et al., 1995), which was not given significant attention (only one exercise was carried out) in our jumping program (Figure 1). Furthermore, during the sprint start, acceleration from 0 velocity to 5–7 m/s is in two to three steps (Mann, 2011). Thus, to achieve this in athletes in the first 15 meters a large number of sprints probably have to be performed before a significant enhancement is recorded. In this study, participants only trained 12 times with, at maximum, 5 to 8 starts per session, with no feedback on technique. This was probably not sufficient to improve sprint times over the first 15m. Despite the importance of sprint technique for speed enhancement (Plisk et al., 2000), this was not a routine practice for the sample group of youth soccer players. In fact, although sprint, acceleration, and changes in direction are movements which are inherent in performance for soccer players in matches and competitions, these were not sufficient to produce significant changes in the first 15m. These results, however, contrast with those observed by Thomas et al. (2009). Indeed, these authors failed to observe any significant differences (p>0.05) in sprint times (5, 10, 14, and 20m) after 6 weeks of plyometric training in adolescent soccer players. Some of these findings can be attributed to a number of factors, especially the specificity of the resistance training regimen. Nevertheless, the participants recruited by Thomas et al. (2009) were randomly assigned to a depth-jump training group or a CMJ protocol with no performance of sprint exercises. Curiously, Jovanovic et al. (2011) and Tønnessen et al. (2011) used a training program which was similar to that used in the present study and found significant changes (p<0.05) in sprinting performance, and therefore, provide support for our experimental results. Because the participants in the present study trained twelve hours per week on average, a slight concern was raised as to whether this sprint program, conducted twice a week, would be sufficient to improve sprint performance over a short period of time.

Regarding kicking performance, in this study an increase of 6.6% in ball velocity was found in the experimental group, which was approximately the same (7.1%) as the findings of Sedano et al. (2009) and Campo et al. (2009) for female soccer players following a 6-week plyometric training program. In this study, an increase of 3.3% was also recorded for the control group. The difference between the groups was not significant but the improved kicking velocity of the training group was probably due to enhanced power of the leg muscles. In a study carried out by Sedano et al. (2009) and Campo et al. (2009), the control group did not show any increase in kicking velocity. However, it showed higher pretest kicking velocities than the training group. In addition, these changes may be the result of an adaptation to the kicking movement following gains in strength and may be the result of an altered stretched-shortening cycle of the musculature involved (Manolopoulos et al., 2006). However, in the current study there were no such findings, while improvements in kicking velocity could be caused by an increased transfer of energy from proximal to distal segments, which may have contributed to higher ball-speed following the plyometric training intervention program. Furthermore, participants are youth players who are still developing and as a result of training they probably improved their soccer kicking performance.

Most studies evaluating the effects of plyometric training use drop jumps (Thomas et al., 2009), hurdle jumps and long horizontal jumps, which can be extremely taxing on the neuromuscular system (Thomas et al., 2009; Michailidis et al., 2012). In this study, vertical jumps, which are not very taxing on the lower limbs, were mainly used. Furthermore, total training time equaled only 15 minutes per session, which is easy to incorporate in regular soccer training undertaken twice a week. This is one advantage of this type of a training program. However, a combined plyometric and sprint program was used for the enhancement of different motor skills in youth soccer players. This makes it difficult to say whether only plyometrics, sprinting, or a combination of both training forms would help in enhancing these motor skills to the greatest degree. In future studies, plyometric or sprint training should be used separately to investigate the effect of these programs on different motor skills in youth soccer players. It is important to determine this before stating what exactly (plyometric, sprinting or a combination) enhances such performances.

## Practical Applications

On the basis of the findings of the present study, it may be concluded that a short 6-week period of combined sprint and plyometric training can significantly improve explosive strength in youth competitive soccer players, and, more importantly, that this improvement can be transferred to soccer kicking performance in terms of ball velocity. Therefore, male soccer professionals can benefit from plyometric training by increasing their ability to use explosive strength effectively while performing a specific activity. However, soccer coaches should also be aware that plyometric training should be combined with regular soccer training in order to ensure that gains in terms of explosive strength are transferred to the kinematic parameters of the kicking movement.

## Figures and Tables

**Table 1:** Training program showing total repetitions per training sessions

**Exercise**	**Training sessions**					
	**1**	**2**	**3**	**4**	**5**	**6**

2-legged jumps	3 × 20	3 × 20	3 × 20	3 × 25	3 × 25	3 × 25
2-legged jumps (knees bent)	3 × 10	3 × 10	3 × 10	3 × 10	4 × 10	4 × 10
Short, quick hops on one leg	3 × 10	3 × 10	3 × 10	3 × 10	2 × 10	2 × 10
1-legged jumps as high as possible	2 × 8	2 × 8	2 × 8	2 × 8	3 × 8	3 × 8
Sprint from a standing position	5 × 20m	6 × 20m	6 × 20m	6 × 20m	2 × 4 × 20m	-
Sprint from a lying position						2 × 4 × 10m

**Exercise**	**Training sessions**					
	**7**	**8**	**9**	**10**	**11**	**12**

2-legged jumps	3 × 30	3 × 30	-	-	-	-
2-legged jumps up steps	-	-	4 × 20	4 × 20	5 × 20	5 × 20
2-legged jumps as far as possible (knees bent)	3 × 10	3 × 10	4 × 10	4 × 10	4 × 10	4 × 10
Short, quick hops on one leg	3 × 10	3 × 10	3 × 10	3 × 10	3 × 10	3 × 10
1-legged jumps as high as possible	3 × 10	3 × 10	-	-	-	-
Heading without ball	-	-	3 × 5	3 × 5	3 × 5	3 × 5
Sprint from lying start position	5 × 30m	5 × 15m	-	-	-	-
Sprint from 5m sideways start	-	-	6 × 30m	6 × 15m	2 × 4 × 30m	2 × 4 × 15m

## References

[b1-jhk-39-157] Alves JMVM, Rebelo AN, Abrantes C, Sampaio J (2010). The Short term effects of complex and contrast training on soccer players’ vertical jump, sprint, and agility abilities. J Strength Cond Res.

[b2-jhk-39-157] Andersen TB, Dorge HC (2011). The influence of speed of approach and accuracy constraint on the maximal speed of the ball in soccer kicking. Scand J Med Sci Sports.

[b3-jhk-39-157] Billot M, Martin A, Paizis C, Cometti C, Babault N (2010). The Effects of an electro-stimulation training program on strength, jumping, and kicking capacities in soccer players. J Strength Cond Res.

[b4-jhk-39-157] Campo S, Vaeyens R, Philippaerts RM, Redondo J, de Benito A, Cuadrado G (2009). The Effects of lower-limb plyometric training on body composition, explosive strength, and kicking speed in female soccer players. J Strength Cond Res.

[b5-jhk-39-157] Chamari K, Chaouachi A, Hambli M, Kaouech F, Wisloff U, Castagna C (2008). The five-jump test for distance as a field test to assess lower-limb explosive power in soccer players. J Strength Cond Res.

[b6-jhk-39-157] Chelly MS, Fathloun M, Cherif N, Ben Amar M, Tabka Z, Van Praagh E (2009). The Effects of a back-squat training program on leg power, jump, and sprint performances in junior soccer players. J Strength Cond Res.

[b7-jhk-39-157] Chimera NJ, Swanik KA, Swanik CB, Straub SJ (2004). The Effects of plyometric training on muscle-activation strategies and performance in female athletes. J Athletic Train.

[b8-jhk-39-157] Christou M, Smilios I, Sotiropoulos K, Volaklis K, Pilianidis T, Tokmakidis SP (2006). The Effects of resistance training on the physical capacities of adolescent soccer players. J Strength Cond Res.

[b9-jhk-39-157] Cometti G, Maffiuletti NA, Pousson M, Chatard JC, Maffulli N (2001). The Isokinetic strength and anaerobic power of elite, sub-elite and amateur French soccer players. Int J Sports Med.

[b10-jhk-39-157] Davids K, Glazier P, Araujo D, Bartlett R (2003). Movement systems as dynamic systems: the functional role of variability and its implications for sports medicine. Sports Med.

[b11-jhk-39-157] Diallo O, Dore E, Duche P, Van Praagh E (2001). The Effects of plyometric training followed by a reduced training programme on physical performance in prepubescent soccer players. J Sports Med Phys Fitness.

[b12-jhk-39-157] Haff GG, Withley A, Potteiger JA (2001). A brief review: Explosive exercises and sports performance. J Strength Cond Res.

[b13-jhk-39-157] Hoff J, Helgerud J (2004). Endurance and strength training for soccer players: physiological considerations. Sports Med.

[b14-jhk-39-157] Jovanovic M, Sporis G, Omrcen D, Fiorentini F (2011). The Effects of speed, agility and quickness training method on power performance in elite soccer players. J Strength Cond Res.

[b15-jhk-39-157] López-Segovia M, Marques MC, van den Tillaar R, González-Badillo JJ (2011). Relationships Between Vertical Jump and Full-Squat Power Outputs With Sprint Times in U21 Soccer Players. J Human Kinetics.

[b16-jhk-39-157] Mann R (2011). The Mechanics of Sprinting and Hurdling.

[b17-jhk-39-157] Manolopoulos E, Papadopoulos C, Salonikidis K, Katartzi E, Poluha S (2004). Strength training effects on physical conditioning and instep kick kinematics in young amateur soccer players during the preseason. Percept Mot Skills.

[b18-jhk-39-157] Michailidis Y, Fatouros IG, Primpa E, Michailidis C, Avloniti A, Chatzinikolaou A, Barbero-Alvarez JC, Tsoukas D, Douroudos II, Draganidis D, Leontsini D, Margonis K, Berberidou F, Kambas A (2012). Plyometrics and Trainability in Pre-Adolescent Soccer Athletes. J Strength Cond Res.

[b19-jhk-39-157] Mújica I, Santiesteban J, Castagna C (2009). The In-season effect of short-term sprint and power training programs on elite junior soccer players. J Strength Cond Res.

[b20-jhk-39-157] Perez-Gomez J, Olmedillas H, Delgado-Guerra S, Ara I, Vicente-Rodriguez G, Ortiz RA, Chavarren J, Calbet JA (2008). The Effects of weight-lifting training combined with plyometric exercises on physical fitness, body composition, and knee extension velocity during kicking in football. Appl Physiol Nutr Metab.

[b21-jhk-39-157] Plisk SS, Baechle TR, Earle RW (2000). Speed, Agility, and Speed-Endurance Development. Essentials of Strength Training and Conditioning.

[b22-jhk-39-157] Requena B, Gonzalez-Badillo JJ, de Villareal ES, Ereline J, Garcia I, Gapeyeva H, Paasuke M (2009). Functional performance, maximal strength, and power characteristics in isometric and dynamic actions of lower extremities in soccer players. J Strength Cond Res.

[b23-jhk-39-157] Sedano S, Vaeyens R, Philippaerts RM, Redondo JC, Cuadrado G (2009). The Anthropometric and anaerobic fitness profile of elite and non-elite female soccer players. J Sports Med Phys Fitness.

[b24-jhk-39-157] Sedano S, Vaeyens R, Philippaerts RM, Redondo JC, de Benito AM, Cuadrado G (2009). The Effects of lower-limb plyometric training on body composition, explosive strength, and kicking speed in female soccer players. J Strength Cond Res.

[b25-jhk-39-157] Thomas K, French D, Hayes PR (2009). The effect of two plyometric training techniques on muscular power and agility in youth soccer players. J Strength Cond Res.

[b26-jhk-39-157] Tønnessen E, Shalfawi SAI, Haugen T, Enoksen E (2011). The effect of 40-m repeated sprint training on maximum sprinting speed, repeated sprint speed endurance, vertical jump, and aerobic capacity in young elite male soccer players. J Strength Cond Res.

[b27-jhk-39-157] Thorlund JB, Aagaard P, Madsen K (2009). Rapid muscle force capacity changes after soccer match play. Int J Sports Med.

[b28-jhk-39-157] Vera JC, Alvarez JG, Medina MM (2008). The Effects of different practice conditions on acquisition, retention, and transfer of soccer skills by 9-year-old schoolchildren. Percept Mot Skills.

[b29-jhk-39-157] de Villarreal ES, Gonzalez-Badillo JJ, Izquierdo M (2008). Low and moderate plyometric training frequency produces greater jumping and sprinting gains compared with high frequency. J Strength Cond Res.

[b30-jhk-39-157] Wisloff U, Castagna C, Helgerud J, Jones R, Hoff J (2004). Strong correlation of maximal squat strength with sprint performance and vertical jump height in elite soccer players. Br J Sports Med.

[b31-jhk-39-157] Wisloff U, Helgerud J, Hoff J (1998). Strength and endurance of elite soccer players. Med Sci Sports Exerc.

[b32-jhk-39-157] Zatsiorsky VM (1995). Science and Practice of Strength Training.

